# Supplementary material for: Nurse, Give Me the News! Understanding Support for and Opposition to a COVID-19 Health Screening System
Source: Int J Environ Res Public Health. 2023 Jan 9;20(2):1164. doi: 10.3390/ijerph20021164 (PMC9859575; doi:10.3390/ijerph20021164)
Supplement: Supplementary file 1 [file ijerph-20-01164-s001.zip › ijerph-2090851-supplementary.pdf]

DEFINITION	CODE	A	B	C	D (> 57.5)	D2 (< 57.5)	F	T-TEST BETWEEN GROUPS
Staff or visitor's capacity to "read" the system correctly (i.e. understand how to interact) as they encounter it.	*Inconsistent with other services*	0	0	0	0	0	6	0.177807808
	System coherence	3	0	1	3	0	1	0.344046032
Extent to which the information communicated by the system is interpretable, useful and necessary for the user.	*Information overload*	2	2	0	0	1	6	0.228268707
	Information organization	11	4	6	10	9	17	0.206349869
	Information visibility	5	1	4	0	3	3	0.79332084
	Visual communication	1	0	0	1	1	0	1
	Situationally responsive	1	0	0	1	2	2	0.025721421
Processes through which the system is able to encourage the user towards correct and efficient use during human-system interactions.	*Learnability of system*	0	1	1	0	0	5	0.277120841
	System is dependent on digital literacy	0	0	0	0	0	4	0.177807808
	System is easy to use	11	4	4	2	1	0	0.185755085
	Informative	3	1	1	1	0	1	0.284606331
Aspects of the system designed to assist the user generally, or specifically serve to increase the knowledge, capability or autonomy of the user.	*System does not facilitate autonomy*	1	0	0	1	0	3	0.380506073
	System shortcuts	3	1	2	6	1	6	0.833508807
	Lack of error recovery	2	0	0	1	1	2	0.391075888
	Independent self-service	0	0	0	1	1	0	0.6328125
	Accessible (mobile)	1	0	1	0	0	1	1
	Quick engagement with system	3	0	2	0	1	1	0.835026074
Considerations and concerns raised by users that refers specifically to the ways in which the system was integrated into the hospital environment.	*Lack of feedback loop*	0	0	0	0	0	4	0.177807808
	Deployment communication	0	0	1	0	1	6	0.108214925
Reliability issues with the system's hardware and their physical and tangible consequences.	*Ergonomic troubles*	0	1	0	0	0	3	0.276207795
	Congestion on site	0	0	1	2	0	3	0.555073152
	Time consuming	0	0	1	2	2	20	0.138918524

	Reliability of system	2	1	4	4	2	8	0.355232431
The "accuracy" dimension of information provided to the system. Reflects the shift in power from manual screening (in which a nurse or staff collects the screening data) to a self-service system (whereby users are responsible for the accuracy of their information) and the implications of this transition.	*Reliability of collected data*	1	0	0	0	1	3	0.071432473
	Inaccuracy of system information	0	0	1	3	1	5	0.284606331
	Privacy	0	0	1	0	0	1	0.6328125
	Retaining the humanity of staff	0	0	0	1	1	3	0.071432473
	Necessity of system	1	1	0	1	0	15	0.216830705

m	BH Corrected p value 0.05	BH at corrected 0.25	Is p value < BH corrected p value for	comparison at 0.25
28	0.001724	0.00862		sig
28	0.003448	0.01724		sig
28	0.005172	0.02586		sig
28	0.006897	0.03448		sig
28	0.008621	0.0431		sig
28	0.010345	0.05172		sig
28	0.012069	0.06034		non-sig
28	0.013793	0.06897		non-sig
28	0.015517	0.07759		non-sig
28	0.017241	0.08621		non-sig
28	0.018966	0.09483		non-sig
28	0.02069	0.10345		non-sig
28	0.022414	0.11207		non-sig
28	0.024138	0.12069		non-sig

28	0.025862	0.12931		non-sig
28	0.027586	0.13793		non-sig
28	0.02931	0.14655		non-sig
28	0.031034	0.15517		non-sig
28	0.032759	0.16379		non-sig
28	0.034483	0.17241		non-sig
28	0.036207	0.18103		non-sig
28	0.037931	0.18966		non-sig
28	0.039655	0.19828		non-sig
28	0.041379	0.2069		non-sig
28	0.043103	0.21552		non-sig
28	0.044828	0.22414		non-sig
28	0.046552	0.23276		non-sig
28	0.048276	0.24138		non-sig
	0.05	0.25		non-sig
